# P32 Therapeutic dilemma due to gut complications with spondyloarthritis

**DOI:** 10.1093/rap/rkac067.032

**Published:** 2022-09-28

**Authors:** Sujata Ganguly, Dan Rogers, Arumugam Moorthy

**Affiliations:** University Hospitals of Leicester NHS Trust, Leicester, United Kingdom; University Hospitals of Leicester NHS Trust, Leicester, United Kingdom; University Hospitals of Leicester NHS Trust, Leicester, United Kingdom

## Abstract

**Introduction/Background:**

Tumor necrosis factor (TNF) inhibitor therapy is one of the primary modalities of treating spondyloarthritis. However, infections secondary to biologic use is a common complication. Clostridium difficile is a gut colonizer which may become pathogenic in the presence of dysbiosis and antibiotic use. Symptomatic clostridium difficile infection(CDI) due to adalimumab(ADA) use causing lymphocytic colitis(LC) is rarely reported. We present a case of axial plus peripheral spondyloarthritis on adalimumab who developed CDI and presented a therapy related dilemma.

**Description/Method:**

We present a case of a 52-year-old lady who was diagnosed as HLAB27 positive axial spondyloarthritis with bilateral sacroiliitis on MRI bilateral sacroiliac joint in 2016. She failed two non-steroidal antinflammatory drugs (NSAID) with high disease activity scores(BASDAI 8.6, BASFI 6.2, Spinal VAS 9). She was eventually initiated on injection adalimumab in 2017. There was clinical improvement after 6 months of initiating ADA. Repeat MRI spine and SI joint showed resolution of inflammatory changes with adalimumab. Sulfasalazine was added in the treatment protocol due to peripheral arthralgia, however she developed a drug rash to it and it was stopped. In September 2021, she developed recurrent non-bloody watery diarrhoeal episodes. Adalimumab was stopped and she tested positive for Clostridium difficile on three occasions. She received multiple courses of vancomycin, fidoxamycin and metronidazole with partial resolution of diarrhoea. Adalimumab was rechallended in January 2022 since her disease activity was worsening in the absence of NSAID and biologic use. However, it was paused again due to worsening of diarrhoea. Gastroenterology conducted a Colonoscopy and biopsy of colonic polyps which showed increased number of intraepithelial lymphocytes with no thickening of subepithelial collagen plate suggestive of lymphocytic colitis (LC). It was postulated that CDI may have been the trigger for her LC. She has been started on local corticosteroid therapy(budesonide) for her LC. At this junction, we were faced with the decision of restarting adalimumab for her which may have been the original cause of her CDI.

**Discussion/Results:**

We present this case in view of a dilemma regarding which problem occurred first Did immunosuppression due to adalimumab lead to CDI? Literature is not suggestive of increased risk of CDI with adalimumab, especially in a non-inflammatory bowel disease (IBD) background. Was the lymphocytic colitis triggered by the CDI or was clostridium difficile just incidentally detected on a background of LC? There is a well documented associated of LC with autoimmune conditions and use of NSAIDs along with proton pump inhibitor is another trigger for it. TNF alpha inhibitor is one of the treatment modalities of refractory LC. Would it be appropriate to restart ADA for her since she responded well despite there being a risk of recurrence of a CDI? Finally, what is the role of gut dysbiosis in the role of CDI and LC in this situation. Presently, she is planned to be treated with local steroid therapy for her LC. ADA will be rechallenged once her gut has responded to the steroid course. In the event of her gut worsening on a rechallenge, she will be given a trial of Infliximab.

**Key learning points/Conclusion:**

The case highlights the crucial point of thinking of causes of diarrhea apart from well documented conditions of ulcerative colitis/Crohn’s disease in an immunosuppressed spondyloarthritis patient. A detailed stool examination including bacterial and parasitic examination is crucial in recurrent diarrhea. The threshold for colonoscopy should be low when episodes are refractory to treatment. The case-based conference gives us an opportunity to receive feedback regarding further management approaches in this case.

